# Liraglutide improves liver microvascular dysfunction in cirrhosis: Evidence from translational studies

**DOI:** 10.1038/s41598-017-02866-y

**Published:** 2017-06-12

**Authors:** Fernanda Cristina de Mesquita, Sergi Guixé-Muntet, Anabel Fernández-Iglesias, Raquel Maeso-Díaz, Sergi Vila, Diana Hide, Martí Ortega-Ribera, José Luís Rosa, Juan Carlos García-Pagán, Jaime Bosch, Jarbas Rodrigues de Oliveira, Jordi Gracia-Sancho

**Affiliations:** 1 Liver Vascular Biology Research Group, Barcelona Hepatic Hemodynamic Lab, IDIBAPS Biomedical Research Institute - CIBEREHD, Barcelona, Spain; 2 University of Barcelona Medical School, Barcelona, Spain; 3 Laboratório de Biofísica Celular e Inflamação, PUCRS, Porto Alegre, RS Brazil; 4 Departament de Ciències Fisiològiques, IDIBELL, Universitat de Barcelona, L’Hospitalet de Llobregat, Barcelona, Spain

## Abstract

Hepatic stellate cells (HSC) play a key role in the development of chronic liver disease (CLD). Liraglutide, well-established in type 2 diabetes, showed anti-inflammatory and anti-oxidant properties. We evaluated the effects of liraglutide on HSC phenotype and hepatic microvascular function using diverse pre-clinical models of CLD. Human and rat HSC were *in vitro* treated with liraglutide, or vehicle, and their phenotype, viability and proliferation were evaluated. In addition, liraglutide or vehicle was administered to rats with CLD. Liver microvascular function, fibrosis, HSC phenotype and sinusoidal endothelial phenotype were determined. Additionally, the effects of liraglutide on HSC phenotype were analysed in human precision-cut liver slices. Liraglutide markedly improved HSC phenotype and diminished cell proliferation. Cirrhotic rats receiving liraglutide exhibited significantly improved liver microvascular function, as evidenced by lower portal pressure, improved intrahepatic vascular resistance, and marked ameliorations in fibrosis, HSC phenotype and endothelial function. The anti-fibrotic effects of liraglutide were confirmed in human liver tissue and, although requiring further investigation, its underlying molecular mechanisms suggested a GLP1-R-independent and NF-κB-Sox9-dependent one. This study demonstrates for the first time that liraglutide improves the liver sinusoidal milieu in pre-clinical models of cirrhosis, encouraging its clinical evaluation in the treatment of chronic liver disease.

## Introduction

Glucagon-like peptide-1 (GLP-1) receptor agonists (GLP-1RA) are a new class of anti-diabetic medications that mimic the effects of incretin hormones^1^. As an incretin hormone, which is synthesized in response to food intake, GLP-1 can stimulate insulin release by pancreatic β-cells in a glucose-dependent manner and suppress glucagon secretion from α-cells^2^. The favourable actions of GLP-1 on glucose homeostasis are mediated through GLP-1 receptors. However, native GLP-1 is rapidly degraded in circulation^3^. Liraglutide, a synthetic GLP-1RA that shares 97% homology with the structure of human GLP-1, possesses a much longer circulating half-life, thereby making it a novel anti-diabetic drug suitable for once-daily injection^1^. Apart from the pancreatic islets, GLP-1 receptors are present in many other tissues and, although its expression within the liver is not clear^4, 5^, recent studies demonstrated efficacy of GLP-1RA in liver diseases, such as NAFLD^6, 7^. In this regard, studies showed other beneficial properties for this type of drugs, including anti-inflammatory and anti-oxidant^8, 9^, which are also important for the resolution of chronic liver disease (CLD).

Cirrhosis is the end stage of CLD that starts with deregulations in the phenotype of all hepatic cells leading to parenchymal and sinusoidal dysfunction^10^. In CLD, both architectural alterations of the liver parenchyma and sinusoidal microvascular dysfunction contribute to the development of portal hypertension^11^. Architectural distortion of the cirrhotic liver is mainly due to excessive synthesis and deposition of extracellular matrix performed by deregulated fibrogenic cells mainly hepatic stellate cells (HSC)^12^. Indeed, in response to liver injury, HSC gradually transdifferentiate to an activated α-SMA-positive phenotype with extensive proliferation, and high vasoconstrictive and pro-inflammatory properties^13, 14^. It is widely accepted that activation of HSC is a key factor in the pathogenesis of liver fibrosis, CLD and portal hypertension^15^. Moreover, an intimate crosstalk between HSC and other sinusoidal cells further contributes to the development and aggravation of CLD^16^.

CLD may improve in response to injury cessation, blockade of pro-fibrogenic mediators or drug-induced HSC inactivation^17^. Unfortunately, current treatment options for CLD and its main complication portal hypertension are limited, and importantly there is no effective therapy available to efficiently ameliorate the hepatic microcirculation of CLD^18^. Therefore, novel therapeutic strategies based on EMA/FDA approved drugs with no systemic adverse effects are required to improve treatments for patients with CLD.

The primary purpose of the present study was to evaluate the effects of liraglutide on HSC phenotype, liver microvascular function and underlying mechanisms in pre-clinical models of CLD.

## Results

### Liraglutide improves the phenotype of Hepatic Stellate Cells

**Figure 1:**
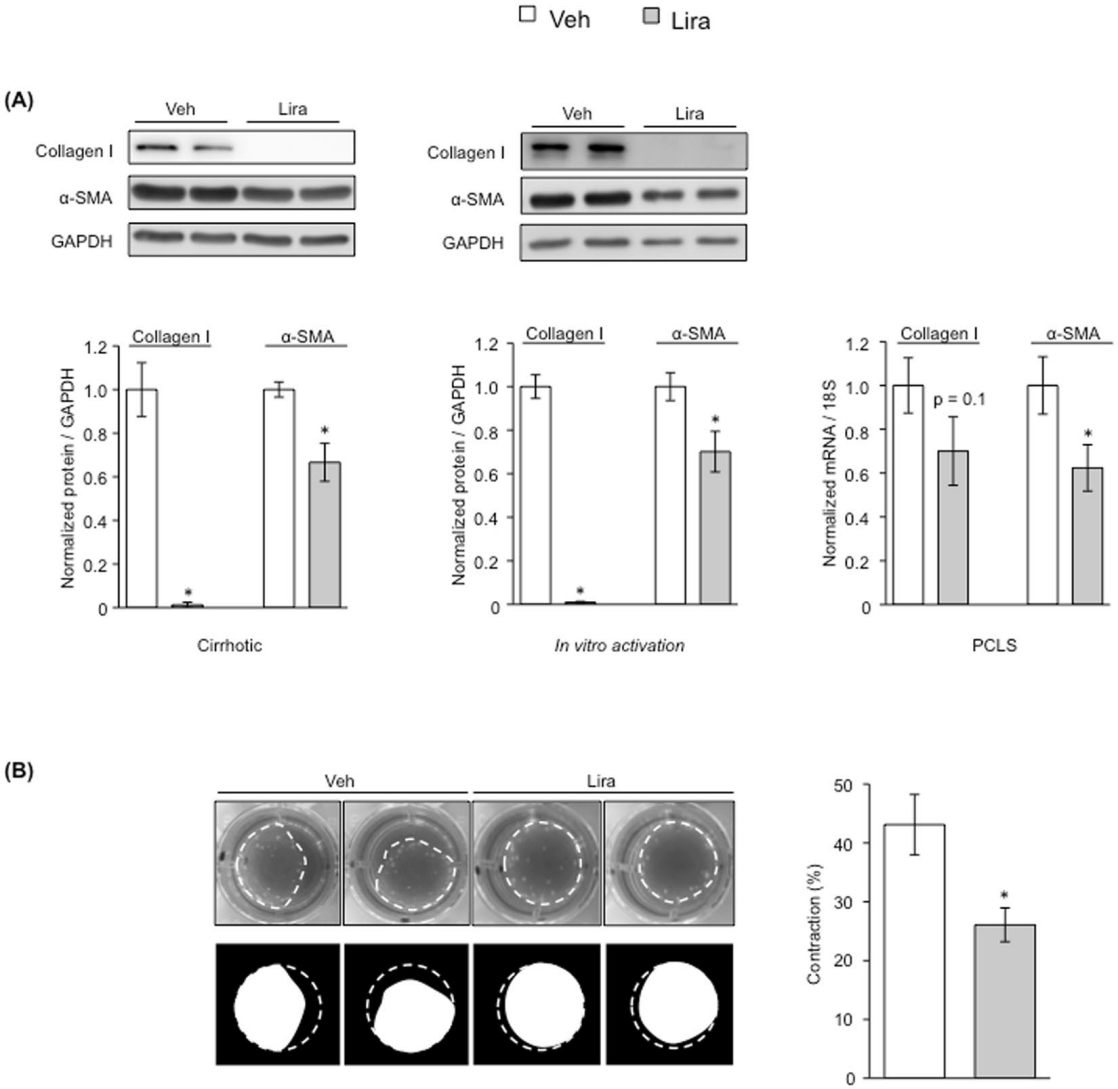
Amelioration of human primary HSC in response to liraglutide. **(A)** Expression of depicted proteins/genes after *in vitro* treatment with 50 µM liraglutide or vehicle in: 1-HSC isolated from cirrhotic human livers (left), 2-quiescent human HSC undergoing *in vitro* activation (middle), and 3-human precision-cut liver slices (PCLS) (right). **(B)** Effects of liraglutide, or its vehicle, on the contraction of primary human HSC. n = 3 per experimental condition. *p < 0.05 vs. vehicle.

Effects of liraglutide on HSC phenotype were assessed in diverse pre-clinical models of CLD. After preliminary dose- and time-response experiments (Supplementary Fig. 1), we characterized liraglutide’s effects promoting the de-activation of cirrhotic primary hHSC and in the prevention of activation of control primary hHSC undergoing 7-day plastic activation. Both conditions showed a marked down-regulation in the activation markers collagen I and α-SMA at a concentration of 50 μM and after 72 h of treatment (Fig. 1A left and middle panels). The anti-fibrotic effects of liraglutide were further validated in human precision-cut liver slices (PCLS) (Fig. 1A right). In addition, the amelioration in hHSC phenotype in response to liraglutide was validated using a functional assay. As shown in Fig. 1B, liraglutide significantly prevented the contraction of primary hHSC.

**Figure 2:**
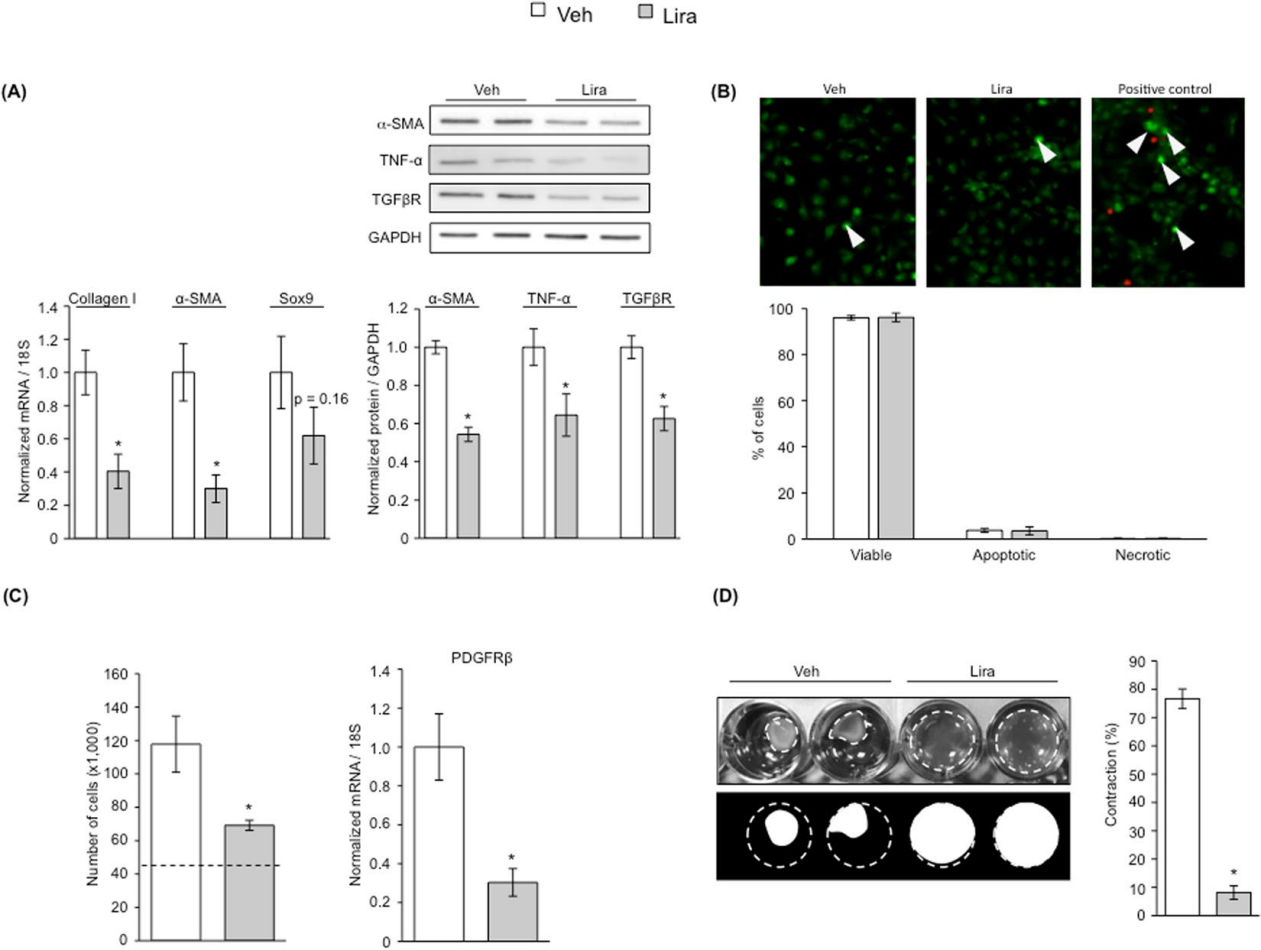
Underlying effects of HSC deactivation due to liraglutide. After 72 h of treatment with 50 µM liraglutide, LX-2 cells were assessed for markers of HSC activation **(A)**, cell viability by double staining with acridine orange (green dense nuclei: apoptosis, indicated by arrowheads) and propidium iodide (red cells: necrosis) **(B)**, HSC proliferation assessed by cell counting and expression of the proliferative marker PDGFRβ **(C)**, and cell contraction **(D)**. n = 3 per experimental condition. *p < 0.05 vs. vehicle.

The effects of liraglutide on activated HSC were further analyzed in LX-2, a widely-accepted human cell line mimicking activated HSC. These experiments indeed showed de-activation of LX-2 cells in response to liraglutide (Fig. 2A), which was associated with significant reductions in the pro-inflammatory and pro-fibrogenic markers TNF-α and TGF-βR1 (Fig. 2A).

Interestingly, LX-2 cells treated with liraglutide showed no significant changes in viability when compared to controls, as observed with the double staining with AO-PI (Fig. 2B). Contrarily, using two different analysis of cell proliferation, the trypan blue exclusion assay and the expression of the proliferative marker PDGFRβ, we herein show the anti-proliferative effects of liraglutide in HSC (Fig. 2C), which were accompanied with a marked reduction in their contraction ability (Fig. 2D). Altogether, validating the global improvement in HSC phenotype in response to liraglutide.

Similar beneficial effects of liraglutide were observed in rat primary HSC (Supplementary Fig. 2).

### Liraglutide improves HSC phenotype and portal hypertension in CLD-rats

**Figure 3:**
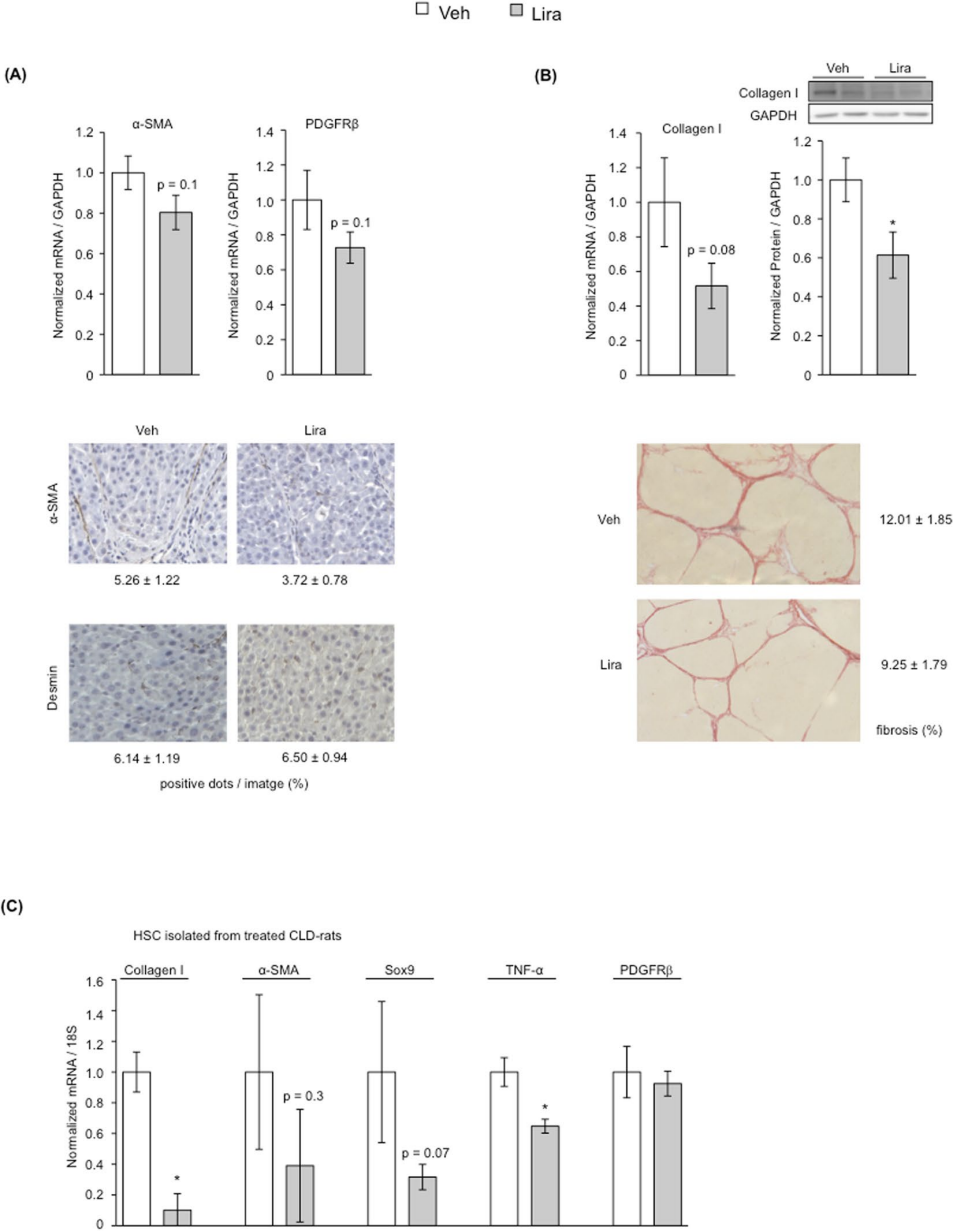
Analysis of HSC phenotype and liver fibrosis in CLD-rats treated with liraglutide. **(A)** Expression of HSC activation markers (α-SMA and PDGFRβ) and Desmin in livers from TAA-CLD-rats treated for 15 days with liraglutide or vehicle. **(B)** Analysis of hepatic fibrosis in rats described in A (collagen I expression and Sirius Red staining). **(C)** Analysis of the phenotype of HSC freshly isolated from rats described in (**A**). *p < 0.05 vs. vehicle. n = 8 (**A** and **B**) and n = 3 (**C**) per group. Results are indicated as mean ± s.e.m.

The potential beneficial effects of liraglutide as a new therapeutic strategy to improve CLD and portal hypertension were also analyzed *in vivo*. After 15 days of treatment, CLD-rats treated with liraglutide displayed lower expression of α-SMA and PDGFRβ (Fig. 3A), accompanied by reductions in extracellular matrix synthesis and deposition as demonstrated by diminished collagen expression and hepatic fibrosis (Fig. 3B). No significant differences in TIMPs and MMPs were observed, thus suggesting that the peak of fibrinolysis already occurred (Supplementary Fig. 3). No effects on HSC viability (desmin expression) were observed, thus supporting the results obtained *in vitro*. Additional analysis of HSC phenotype in cells freshly isolated from CLD-rats treated with liraglutide, or vehicle, confirmed the marked beneficial effects of the drug promoting HSC deactivation (Fig. 3C).

Possible beneficial effects of liraglutide on hepatic and systemic hemodynamics in CLD-rats were also analyzed. Table 1 shows the morphometric and hemodynamic data from these animals. As expected, CLD-rats treated with liraglutide exhibited a slight but significant reduction in body weight, which is in agreement with previous studies^19^. Importantly, liraglutide-treated animals showed significantly lower portal pressure than vehicle-treated animals (11.6 ± 0.8 vs. 9.3 ± 1.0 mmHg; −20%; p = 0.03) without changes in portal blood flow, thus suggesting an improvement in the hepatic vascular resistance (9.5 ± 1.8 vs. 5.7 ± 1.3 mmHg·mL·min^−1^·g^−1^; −23%; p = 0.1). No effects of liraglutide on systemic hemodynamic or biochemical tests were observed.

**Table 1:** Effects of Liraglutide on hepatic and systemic hemodynamics, and biochemical parameters in rats with chronic liver disease due to chronic TAA administration, represented as mean ± s.e.m. PP, portal pressure; MAP, mean arterial pressure; PBF, portal blood flow; HVR, hepatic vascular resistance; AST, aspartate aminotransferase; ALT, alanine aminotransferase; TG, triglycerides; FFA, free fatty acids.

	Vehicle n = 11	Liraglutide n = 11	*p value*
PP (mmHg)	11.6 ± 0.8	9.3 ± 1.0	*0.03*
MAP (mmHg)	99.5 ± 7.2	89.9 ± 7.1	0.4
PBF (mL/min)	11.9 ± 1.0	11.5 ± 2.3	0.5
HVR (mmHg·min·mL^−1^·g^−1^)	9.5 ± 1.8	5.7 ± 1.3	0.1
*ex vivo* HVR (mmHg·min·mL^−1^·g^−1^)	1.6 ± 0.3	0.9 ± 0.04	0.07
Body weight pre-treatment (g)	289 ± 12	288 ± 7	0.5
Body weight post-treatment (g)	310 ± 9	274 ± 9	*0.03*
Liver weight (g)	8.4 ± 0.7	6.7 ± 0.4	0.1
AST (U/L)	105 ± 14	126 ± 14	0.3
ALT (U/L)	61 ± 10	67 ± 4	0.5
Albumin (g/L)	15.3 ± 1.3	16.3 ± 0.5	0.5
Cholesterol (mg/dL)	54.0 ± 8.3	44.6 ± 5.8	0.4
TG (mg/dL)	31.2 ± 5.6	26.8 ± 3.4	0.5
FFA (µmol/L)	506 ± 66	477 ± 55	0.7

**Figure 4:**
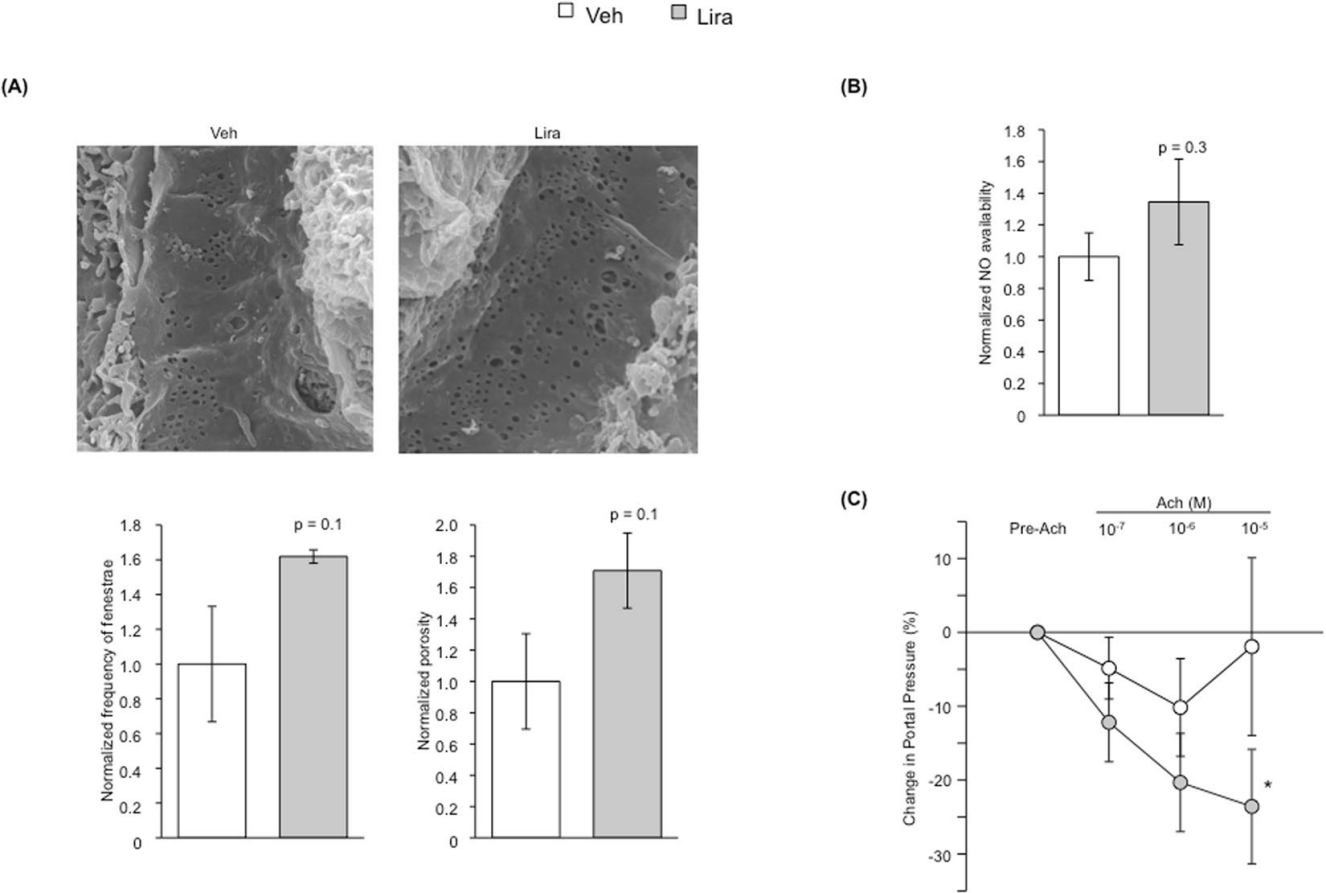
Effects of liraglutide on hepatic endothelial phenotype and microvascular function. **(A)** Liver sinusoidal fenestrae analysis by means of frequency (no. fenestrae/cell area) and porosity (fenestrae area/cell area) in TAA-CLD-rats treated with liraglutide or vehicle. **(B)** Hepatic nitric oxide (NO) bioavailability in rats described in A. **(C)** Hepatic microvascular function, calculated as the decrease in portal pressure in response to increasing doses of the endothelium-dependent vasodilator acetylcholine after vasoconstriction with methoxamine. *p < 0.05 vs. vehicle. n = 3 (**A**), n = 8 (**B**) and n = 5 (**C**) per group.

Intrahepatic microcirculatory amelioration in response to liraglutide was further confirmed analyzing the hepatic microvascular phenotype. Indeed, LSEC from animals receiving liraglutide showed a significant reversal in their capillarization, as suggested by marked increments in fenestrae frequency and porosity (Fig. 4A), and a trend to higher nitric oxide bioavailability (Fig. 4B). In addition, characterization of the hepatic microvascular function *ex vivo* confirmed the global sinusoidal improvement, as demonstrated by reduction in HVR (Table 1) and improved liver vascular response to incremental doses of acetylcholine (Fig. 4C).

### Liraglutide has a complementary effect with simvastatin improving HSC

Treatment of LX-2 cells with liraglutide did not modify the expression of the simvastatin-inducible transcription factor KLF2 (Supplementary Fig. 4 left). However, LX-2 treated with liraglutide or simvastatin showed reduced levels of α-SMA in the same magnitude. Interestingly, when both drugs were combined further reduced α-SMA (Supplementary Fig. 4 right), altogether suggesting that liraglutide has a complementary effect to simvastatin improving the phenotype of activated HSC.

### Liraglutide improves HSC phenotype and liver microcirculation probably through a GLP1-R independent mechanism

Analysis of GLP1-R expression in rat and human liver tissues and HSC showed no detectable mRNA expression (Supplementary Fig. 5A), while a band corresponding to 53 kDa (predicted GLP-1R molecular weight) was only detected in LX-2 and barely present in cirrhotic and NASH human livers, but not in control human or rat livers (either control or cirrhotic) (Supplementary Fig. 5B). Accordingly, analysis of the GLP-1R secondary messenger PKA in rHSC and LX-2 treated with liraglutide did not show differences in its phosphorylation in comparison to cells treated with vehicle (Supplementary Fig. 5C), and incubation of LX-2 with the GLP-1R antagonist Exendin 9–39 did not affect the de-activation effects of liraglutide (Supplementary Fig. 5D). Oppositely, liraglutide did repress the NF-κB molecular pathway (Supplementary Fig. 6).

## Discussion

The major findings of the current study are that liraglutide promotes a marked amelioration in the phenotype of activated HSC, which in a pre-clinical model of chronic liver disease leads to significant improvement in portal hypertension and liver fibrosis. Importantly, the de-activating effects of liraglutide are herein demonstrated in human primary HSC and human liver tissue.

Liraglutide was developed as an anti-diabetic drug predictably acting on GLP-1R in pancreatic β-cells. Interestingly, different studies have demonstrated that these receptors may not be only limited to pancreatic β-cells. Considering the beneficial anti-inflammatory effects of GLP-1R agonists on cardiac fibrosis and NASH^20–22^, we aimed the present study at analyzing the effects of liraglutide in chronic liver disease (CLD).

Liver cirrhosis is the end stage situation of CLD being the main triggering factor a complex multicellular response of all hepatic cells. Indeed, in front of a chronic injury both parenchymal and non-parenchymal cells undergo profound changes in their phenotype, becoming highly de-regulated and ultimately leading to fibrosis and microvascular dysfunction^16^. The most relevant clinical consequence of sinusoidal cells de-regulation is the development of portal hypertension, which derives both from pathological increases in intrahepatic vascular resistance and in portal blood flow. Considering the importance of HSC in CLD progression and aggravation, many studies focused on liver-specific drugs capable of inactivating the HSC, however few studies have advanced to the clinical stage^23^.

Our study is the first showing that liraglutide is able to improve the phenotype of HSC. Indeed, we performed dose- and time-dependent experiments indicating that liraglutide de-activates HSC as demonstrated by reduced expression of α-SMA and collagen. Similarly, we observed prevention of HSC activation in response to liraglutide, therefore suggesting possible beneficial effects of the drug when administered at early stages of CLD. Importantly, we planned this study as a bed to bench-side one, and not vice versa, therefore firstly evaluating the effects of liraglutide in human primary HSC, to latterly use different preclinical models of CLD to further study the molecular mechanisms of such ameliorations.

Hepatic stellate cells are activated in response to different liver injuries, or due to paracrine factors, promoting tissue repair. Their response includes cell mobilization, proliferation, migration towards the lesion, and production of extracellular matrix components. When continuous liver injury occurs, HSC become chronically activated, acquire high expression of pro-inflammatory, pro-fibrogenic and proliferative markers like TNF-α, TGFβ and PDGFRβ, ultimately representing the main cell-type responsible for fibrosis deposition^24, 25^. In the present study, we show that improvement in HSC phenotype in response to liraglutide was accompanied by marked reductions in the expression of these cytokines and proliferation markers, without affecting cell viability. Altogether suggesting that liraglutide promotes the de-activation of HSC, reduces their proliferation but does not induce cell apoptosis. Such anti-inflammatory effects, which are potentially optimal for the resolution of liver fibrosis *in vivo*, are quite different from previous studies showing concomitant de-activation and apoptosis/necrosis of HSC in response to certain therapeutic strategies^26–28^. Importantly, analysis of HSC was not limited to molecular markers, but also included the cell contraction functional assay, which further confirmed the global improvement of HSC phenotype in response to liraglutide.

Additionally, we tested the possible beneficial effects of liraglutide when administered to human liver tissue. Taking advantage of the precision cut liver slices technique, considered an excellent tool to analyze the effects of drugs within the liver^29^, we observed marked reductions in the expression of α-SMA and collagen in response to liraglutide, therefore corroborating the anti-fibrotic effects of the drug.

Once the phenotype of HSC was characterized *in vitro*, we studied the effects of a physiological-relevant dose of liraglutide administered *in vivo*. Liraglutide treatment markedly improved both HSC and LSEC phenotypes. In fact, HSC activation markers collagen I, α-SMA and PDGFRβ were reduced in CLD rats receiving liraglutide, which was accompanied by amelioration in LSEC fenestrae and NO bioavailability. Although we herein demonstrate direct action of liraglutide on HSC, we do not rule out possible paracrine interactions between both sinusoidal cell types in response to the drug^16, 30^. Importantly, global improvement in the sinusoidal phenotype led to regression of liver fibrosis, and to significant amelioration in the hepatic microvascular dysfunction. Indeed, liraglutide was able to reduce the PP in rats with CLD and portal hypertension. Such improvement in hepatic hemodynamics was mostly due to a significant improvement in the intrahepatic microvascular dysfunction, as demonstrated by the estimations of the *in vivo* and *ex vivo* HVR, and the analysis of the *ex vivo* vasodilatory capacity in response to incremental doses of acetylcholine. Importantly, liraglutide did not affect systemic hemodynamics.

We next ascertained which could be the molecular pathway underlying liraglutide effects on HSC, and consequently on liver microcirculation and fibrosis. First, and considering that liraglutide was formulated to act on GLP-1 receptor, we analyzed the expression of this receptor both in HSC isolated from rat and humans, and also in liver tissues. Surprisingly, GLP-1R mRNA was not detected in whole liver homogenates, primary human HSC or LX-2 cells using two different Taqman probes with PCR reactions going up to 60 cycles. At the protein level, western blot of hepatic samples using an antibody against GLP-1R showed signal in LX-2 lysates at the predicted GLP-1R molecular weight, but this signal was barely present in cirrhotic and NASH human tissue, while it was not detected in control human livers or rat liver tissue (either control or cirrhotic). These observations suggest that the beneficial effects of liraglutide in rat HSC and its effects *in vivo* would not be dependent on GLP-1R. Previous reports already showed contradictory results in terms of GLP-1R expression within the liver^4, 5^, and we are not totally convinced that the band detected by the antibody in human samples really corresponds to GLP-1R (as it is contradictory to the two specific mRNA TaqMan probes). In agreement, additional experiments showed lack of protein kinase A phosphorylation (marker of GLP-1R activation)^31, 32^ in response to liraglutide, and no differences in liraglutide-mediated HSC de-activation and proliferation when an antagonist of GLP-1R was used. Secondly, we analyzed the expression of the transcription factor Kruppel-like factor 2 (KLF2) in response to liraglutide since the effects of liraglutide on HSC were quite similar to those previously observed using statins^26, 33–35^. These experiments showed no up-regulation in response to the drug, moreover a synergistic effect of liraglutide and simvastatin de-activating HSC was observed, thus suggesting that they act by different pathways and could be used in combination at the bedside. Lastly, we evaluated the NF-κB molecular pathway, which plays a major role in liver fibrosis^36^ and is inhibited by liraglutide in the endothelium^37^. Interestingly, liraglutide down-regulated the expression of NF-κB, and also of its target gene Sox9.

With these results we cannot delineate the exact molecular mechanism driving liraglutide’s beneficial effects, which represents a limitation of the study. Nevertheless, considering the main role of NF-κB and Sox9 modulating HSC phenotype^38, 39^, and the findings described above, we herein suggest that in the specific scenario of CLD liraglutide may improve HSC phenotype and liver microcirculation through a GLP-1R independent mechanism, being the NF-κB–Sox9 pathway a solid candidate to mediate, at least in part, these effects. Desirable future experiments, out of the scope of the present study, will clarify the specific receptor and the molecular mechanisms that mediate the beneficial effects of this drug in chronic liver disease.

In conclusion, the present study describes the anti-fibrotic effects of liraglutide both in rodent and, importantly, human pre-clinical models of chronic liver disease, therefore encouraging its application at the bedside as new therapeutic tool to improve cirrhosis and portal hypertension. Moreover, the potential results of the LEAN trial opens the possibility to use liraglutide not only to improve mild NASH^7^, but promote regression of advanced chronic liver disease. Desirable future trials will clarify this promising therapeutic alternative.

## Materials and Methods

### Animal models of chronic liver disease (CLD)

The study was carried out in male Wistar rats (Charles River Laboratories, Barcelona, Spain). Animals were kept in environmentally controlled animal facilities. All procedures were approved by the Laboratory Animal Care and Use Committee of the University of Barcelona and were conducted in accordance with the European Community guidelines for the protection of animals used for experimental and other scientific purposes (EEC Directive 86/609). The personnel who prepared and administered treatments and those that performed the experimental studies were different. Treatment’s codes were not open for interpretation of the results until the inclusion of all animals.

#### Induction of CLD by thioacetamide (TAA)

TAA (Sigma Chemical Co) was dissolved in 0.9% normal saline approximately one hour before injection. Treatment groups received 200 mg/kg of TAA twice per week for a total of 12 weeks while control groups received the same volume of 0.9% normal saline^18^.

#### Induction of CLD by Carbon Tetrachloride (CCl_4_)

Rats underwent inhalation exposure to CCl_4_ (Sigma) and received phenobarbital (0.3 g/L) in the drinking water. When rats developed ascites, toxicants administration was stopped^40^.

### HSC isolation, culture and treatments

#### Isolation and culture of HSC

HSC were isolated from human (hHSC: control or cirrhotic) and rat (rHSC: control, TAA-CLD or CCl_4_-CLD) livers as described^26^ with minor modifications. Briefly, liver tissues were perfused with collagenase A, pronase and DNase (all Roche) in Gey’s Balanced Salt Solution (GBSS; Sigma), and dispersed cells were fractionated by density gradient centrifugation using 11.5% Optiprep (Sigma). HSC were cultured in Iscove’s Modified Dulbecco’s Media (IMDM, Invitrogen, Gibco) supplemented with fetal bovine serum, glutamine, antibiotics and amphotericin B. Results using primary HSC derived from at least 3 independent isolations and 3 replicates.

Immortalized human-activated HSC LX-2 were cultured as described^11^. Results using LX-2 derived from at least three replicates per experimental condition.

#### HSC treatments

HSC were incubated with liraglutide (Novo Nordisk), or its vehicle PBS, at different concentrations (1 μM, 10 μM, 50 μM and 100 μM) and for different periods of time (24 h, 48 h and 72 h). *In vitro* dosing of liraglutide was based on previous literature^41^. Considering that liraglutide exerted beneficial effects at 50 μM and after 72 h of treatment, all subsequent experiments were performed using these experimental conditions. Simvastatin was used in 10 µM dose alone and in combination with liraglutide. Exendin fragment 9–39 (Sigma-Aldrich; 10 nM–1 uM) was used as a GLP-1R antagonist^41^.

#### Cell viability and proliferation

Equal number of LX-2 were seeded and after 72 h of liraglutide or vehicle, floating and adhered cells were collected and counted using a hemocytometer with trypan blue dye exclusion (FLUKA).

#### Cell apoptosis

Cells were incubated with fresh medium containing 800 ng/mL Acridine Orange (AO) and 5 μg/mL Propidium Iodide (PI) for 10 min at 37 °C and then washed with PBS. Fresh medium was added and cell death was examined using a fluorescence microscope (Olympus BX51, Tokyo, Japan) equipped with a digital camera (Olympus, DP72). AO is a metachromatic dye that stains both viable and apoptotic cells by intercalating into DNA and emits green fluorescence upon excitation at 480–490 nm. Nevertheless, nuclear condensation that occurs during apoptosis glares a more intense fluorescence. PI is excluded by viable cells but can penetrate cell membranes of dying or dead cells due to necrosis, emitting red fluorescence. Positive controls (*in vitro* ischemia and reperfusion), and negative controls (without dye) were included^26^.

#### Cell contraction

Contraction of HSC was performed as previously described with some modifications^42^. Briefly, culture plates were incubated with 1% BSA-PBS and afterwards filled with a mix of collagen (2 mg/mL) and human HSC (1–2 × 10^5^ cells/mL). Once the gels were solidified, serum free IMDM with 50 µM liraglutide or vehicle was added. After 24 h, contraction was induced by adding 10% FBS for 24 h. Finally, the contraction area was digitalized and measured with ImageJ software. The results are expressed as % of contraction relative to the initial area of the gel.

### Characterization of CLD-rats treated with liraglutide

#### Liraglutide administration

TAA-CLD-rats received by subcutaneous injections, twice a day, either liraglutide (0.5 mg/kg/day; n = 11) or vehicle (0.9% NaCl; n = 11) during 15 days. The dose was selected based on a conversion calculation starting from the dose used in humans and agreed with previous publications^19, 43, 44^. Administration of liraglutide to CLD-rats started one week after stopping the administration of TAA.

#### In vivo hemodynamics

Rats (n = 8 per group) were anesthetized with ketamine hydrochloride (100 mg/Kg; Merial Laboratories) plus midazolam (5 mg/kg; Laboratorios Reig Jofré) intraperitoneally. A tracheotomy was performed and a polyethylene tube PE-240 was inserted into the trachea to ensure a patent airway. PE-50 catheters were introduced into the femoral artery to measure mean arterial pressure (MAP; mmHg) and into ileocolic vein to measure portal pressure (PP; mmHg). A perivascular ultrasonic flow probe (Transonic System) was placed around the portal vein, as close as possible to the liver to avoid portal-collateral blood flow, in order to measure portal blood flow (PBF; mL∙min^−1^). Hepatic vascular resistance (mmHg·min·mL^−1^·g^−1^) was calculated as: PP/PBF. Blood pressures and flows were registered on a multichannel computer based recorder (Power Lab; AD Instruments). Temperature of the animals was maintained at 37 ± 0.5 °C and hemodynamic data were collected after 20 min stabilization^40, 45^. Blood serum and plasma samples were stored for biochemical analysis.

#### Liver microvascular function

Immediately after recording *in vivo* hemodynamics, rat livers were isolated and perfused with Krebs buffer as previously described (n = 5 per group)^45, 46^. The perfused rat liver preparation was allowed to stabilize for 20 min before vasoactive substances were added. Intrahepatic microcirculation was pre-constricted by adding the α1-adrenergic agonist methoxamine (Mtx; 10^−4^ M; Sigma) to the reservoir, and liver microvascular function was assessed as concentration–response curves to cumulative doses of acetylcholine (Ach; 10^–7^–10^−5^ M; Sigma). Liver tissue was snap-frozen for subsequent molecular analysis.

#### Evaluation of hepatic fibrosis

CLD-rat livers were fixed in 10% formalin, embedded in paraffin, sectioned, and stained with 0.1% Sirius Red, photographed, and analyzed using a microscope equipped with a digital camera. The red-stained area was measured using Axiovision software^26^. Values are expressed as the mean of 8 fields per sample.

#### Sinusoidal characterization using Scanning Electron Microscope

In a sub-group of animals (n = 3 per group), after obtaining *in vivo* hemodynamics, livers were perfused through portal vein with a solution containing 2.5% glutaraldehyde and 2% paraformaldehyde and fixed overnight at 4 °C. Samples were washed 3 times with 0.1 M cacodylate buffer. Liver sections were fixed with 1% osmium in cacodylate buffer, dehydrated in ethanol, and dried with hexamethyldisilazane. Six randomly selected blocks from each animal were mounted onto stubs, and sputter coated with gold. 10 images per animal were acquired at a resolution of 15,000x using a Jeol 6380 Scanning Electron Microscope (JEOL Ltd, Tokyo, Japan). Liver sinusoidal fenestrations were quantified using ImageJ Software (NIH)^47^.

#### Nitric Oxide bioavailability

Levels of cGMP, a marker of NO bioavailability, were analyzed in liver homogenates using an enzyme immunoassay (Cayman Chemical Co., Ann Arbor, MI) as previously described^48^.

### RNA isolation and quantitave PCR

RNA from cells and tissue were extracted using RNeasy mini kit (Qiagen) and Trizol (Life Technologies), respectively. RNA quantification was performed using a NanoDrop spectrophotometer. cDNA was obtained using QuantiTect reverse transcription kit (Qiagen). Real-Time PCR were performed in an ABI PRISM 7900HT Fast Real-Time PCR System, using TaqMan predesigned probes for Col1A1 (Hs00164004_m1, Rn01463848_m1), α-SMA (Hs00426835_g1, Rn01759928_g1), PDGFRβ (Hs01019589_m1, Rn01491838_m1), GLP1-R (Hs00157705_m1, Hs01006326_m1), Sox9 (Hs00165814_m1, Rn01751070_m1) and GAPDH or 18 S as endogenous controls. Results, expressed as 2^−ΔΔCt^, represent the x-fold increase of gene expression compared with the corresponding control group.

### Western blot analysis

Cells were rinsed twice with PBS and lysed with Triton lysis buffer. Livers were homogenized in triton-lysis buffer for whole protein extraction. Aliquots from each sample containing equal amounts of protein were run on a sodium dodecylsulphate polyacrylamide gel, and transferred to a nitrocellulose membrane. After the transfer, blots were blocked with Tris buffered saline containing 0.05% Tween-20 and 5% non-fat dry milk or 3% albumin and subsequently incubated overnight at 4 °C with primary antibodies against collagen I (ABT123, Millipore), α-SMA (A2547, Sigma), TNF-α (sc-1351, Santa Cruz Biotechnology), TGFβR (sc-398, Santa Cruz Biotechnology), GAPDH (sc-32233, Santa Cruz Biotechnology), p-PKA (4781, Cell Signaling), NF-κB (6956, Cell Signaling) and IκB (4812, Cell Signaling), all at 1:1000 dilution. Then membranes were incubated with the appropriate horseradish peroxidase-conjugated secondary antibody at room temperature. Protein expression was determined by densitometric analysis using the LAS4000 (GE Healthcare) and Image Studio Lite software (LI-COR). Quantitative densitometric values of all proteins were normalized to GAPDH.

### Precision-cut liver slices of human livers (PCLS)

Fresh human liver biopsies were used to obtain 250 μm slices using a Vibratome VT1000S (Leica Microsystems, Wetzlar, Germany). Samples were washed in PBS, soaked in 4% agarose solution (Ultrapure LMP Agarose, Invitrogen, Carlsbad, California, USA) for 20 min, and then orientated, mounted and immobilized using cyanoacrylate glue. Tissue slices were placed on organotypic tissue culture plate inserts (Millicell®-CM; Millipore). Tissues were maintained at 37 °C in a 5% CO_2_ humidified incubator using 1.1 mL of Williams’ Medium E supplemented with 1% inactivated fetal bovine serum, 2 mM L-Glutamine, 50 U/mL penicillin and 50 μg/mL streptomycin. Tissue slices were incubated with 50 µM liraglutide for 24 h. Sections were then transferred to a 1.5 mL tube and lysed for RNA isolation and qPCR^29^.

### Ethics information

Quiescent hHSC (for prevention of *in vitro* activation) and PCLS were obtained from remnants from partial hepatectomy. Livers were considered control, but exhibited fibrosis staging between F1 and F2. In all cases surgery was recommended to excise tumor metastasis from colon carcinoma. Cirrhotic hHSC (for *in vitro* de-activation) were isolated from remnant cirrhotic livers (all alcoholic aetiology) obtained after transplantation. The Ethics Committee of the Hospital Clinic de Barcelona approved the experimental protocol, samples manipulation and isolation procedures were carried out following good laboratory practices; in all cases patients signed the informed consent.

### Statistical analysis

Statistical analysis was performed with the SPSS V.23.0 for Windows statistical package (IBM, Armonk, New York, USA). Results are expressed as mean ± s.e.m. Normality of samples was assessed using the Kolmogorov Smirnov test. For samples following a normal distribution, comparisons between groups were performed with the Student t test or analysis of variance, followed by a Bonferroni Post Hoc test when adequate. Otherwise, comparisons were assessed with the non-parametric Mann-Whitney U or Kruskal Wallis test when adequate. Differences were considered significant at a *p* value < 0.05.

## Electronic supplementary material

Supplementary info to be published
