# Supplementary material for: Liraglutide improves liver microvascular dysfunction in cirrhosis: Evidence from translational studies
Source: Sci Rep. 2017 Jun 12;7:3255. doi: 10.1038/s41598-017-02866-y (PMC5468330; doi:10.1038/s41598-017-02866-y)
Supplement: Supplementary file 1 — Supplementary info to be published [file 41598_2017_2866_MOESM1_ESM.pdf]

Liraglutide improves liver microvascular dysfunction in cirrhosis: Evidence from translational studies.

Fernanda Cristina de Mesquita, Sergi Guixé-Muntet, Anabel Fernández-Iglesias, Raquel Maeso-Díaz, Sergi Vila, Diana Hide, Martí Ortega-Ribera, José Luís Rosa, Juan Carlos García-Pagán, Jaime Bosch, Jarbas Rodrigues de Oliveira, Jordi Gracia-Sancho

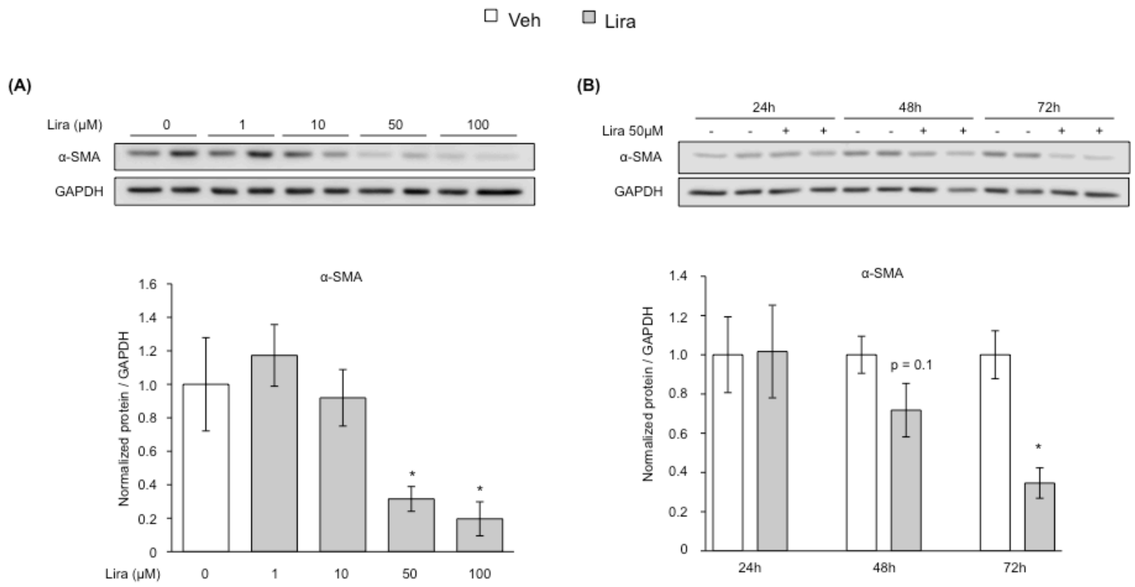

Supplementary figure 1. Analysis of LX-2 de-activation in terms of α-SMA expression in response to liraglutide administered at different doses and times. n=3 per experimental condition. *p<0.05 vs. vehicle.

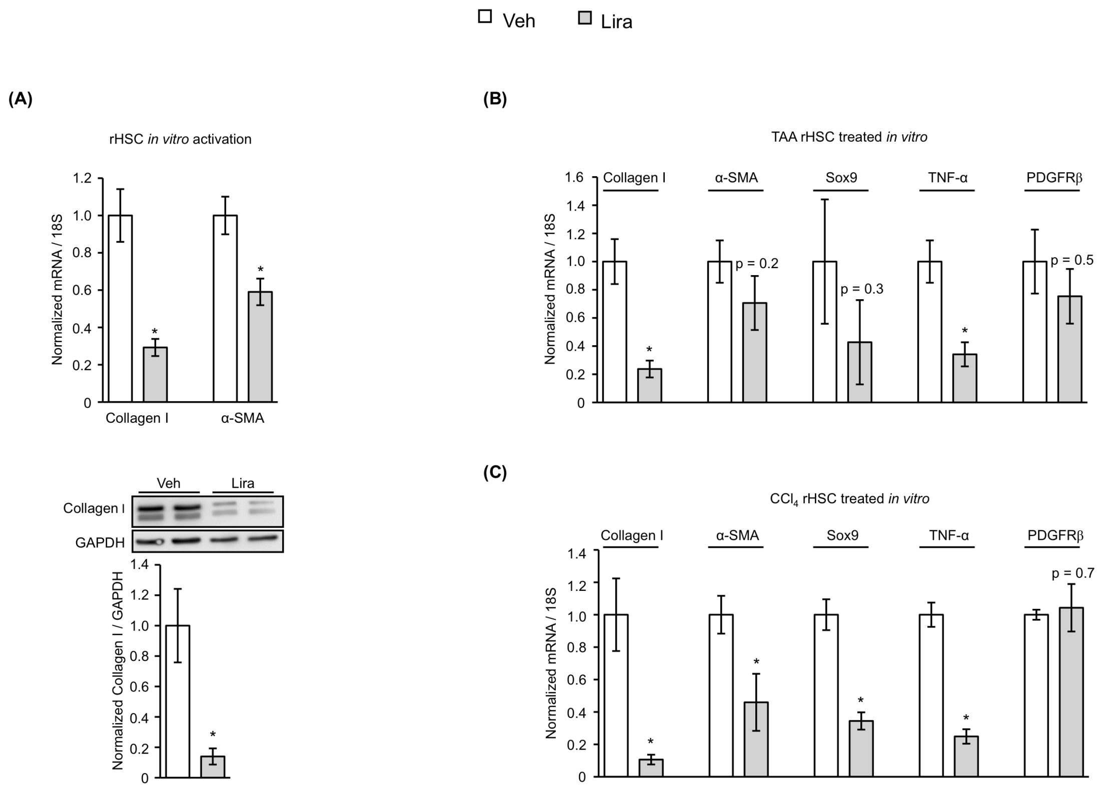

Supplementary figure 2. Effects of 72h-liraglutide on the *in vitro* activation of primary quiescent HSC isolated from healthy rats **(a),** and in the amelioration of activated HSC isolated from TAA-CLD **(b)** and CCl_4-CLD **(c)** rats. n=3 per group. *p<0.05 vs. vehicle.

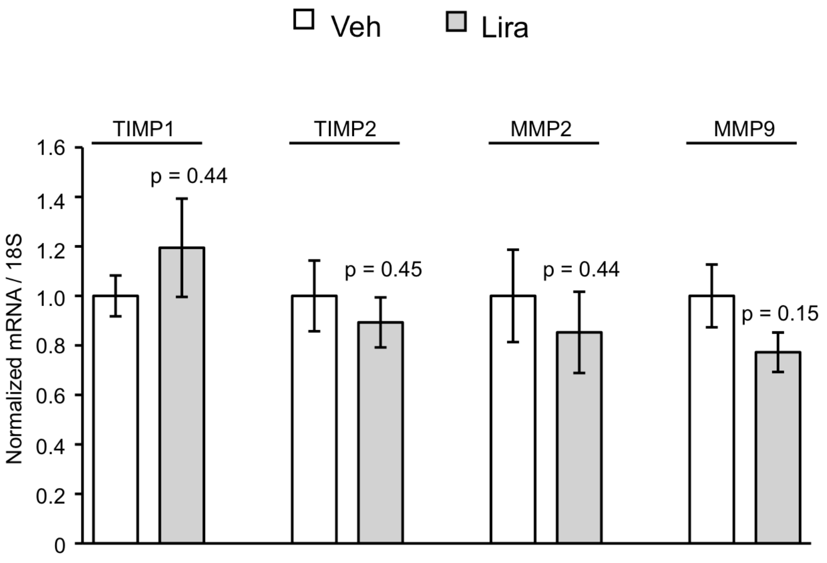

Supplementary figure 3. Expression of TIMPs and MMPs in livers from TAA-CLD-rats treated for 15 days with liraglutide, or vehicle. n=8 per group.

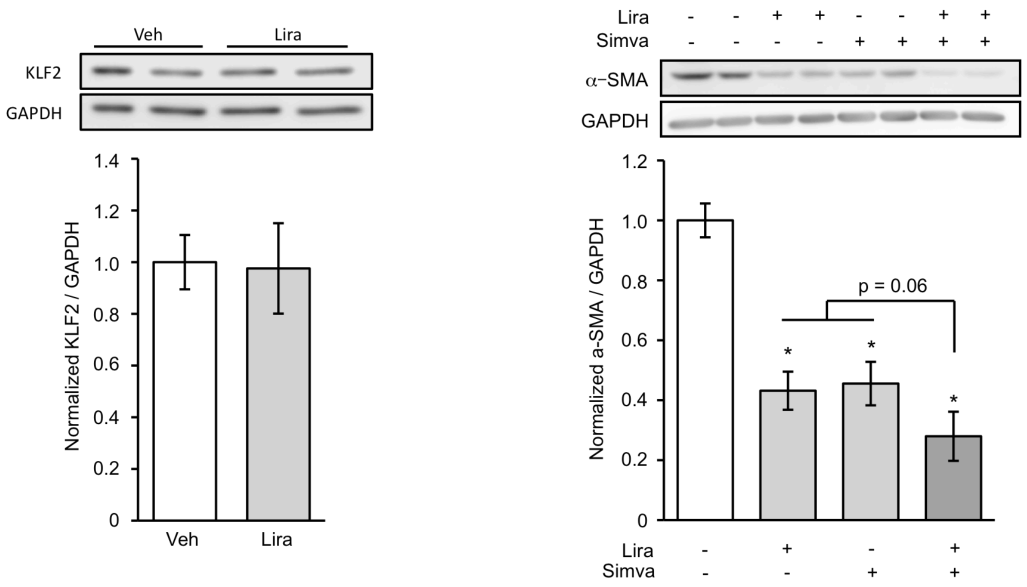

Supplementary figure 4. (a) Expression of the transcription factor KLF2 in LX-2 cells treated with 50 μM liraglutide, or vehicle. **(b)** Expression of α-SMA in LX-2 cells treated with 50 μM liraglutide, or vehicle, in combination with 10μM simvastatin, or its vehicle. n=3 per experimental condition. *p<0.05 vs. vehicle

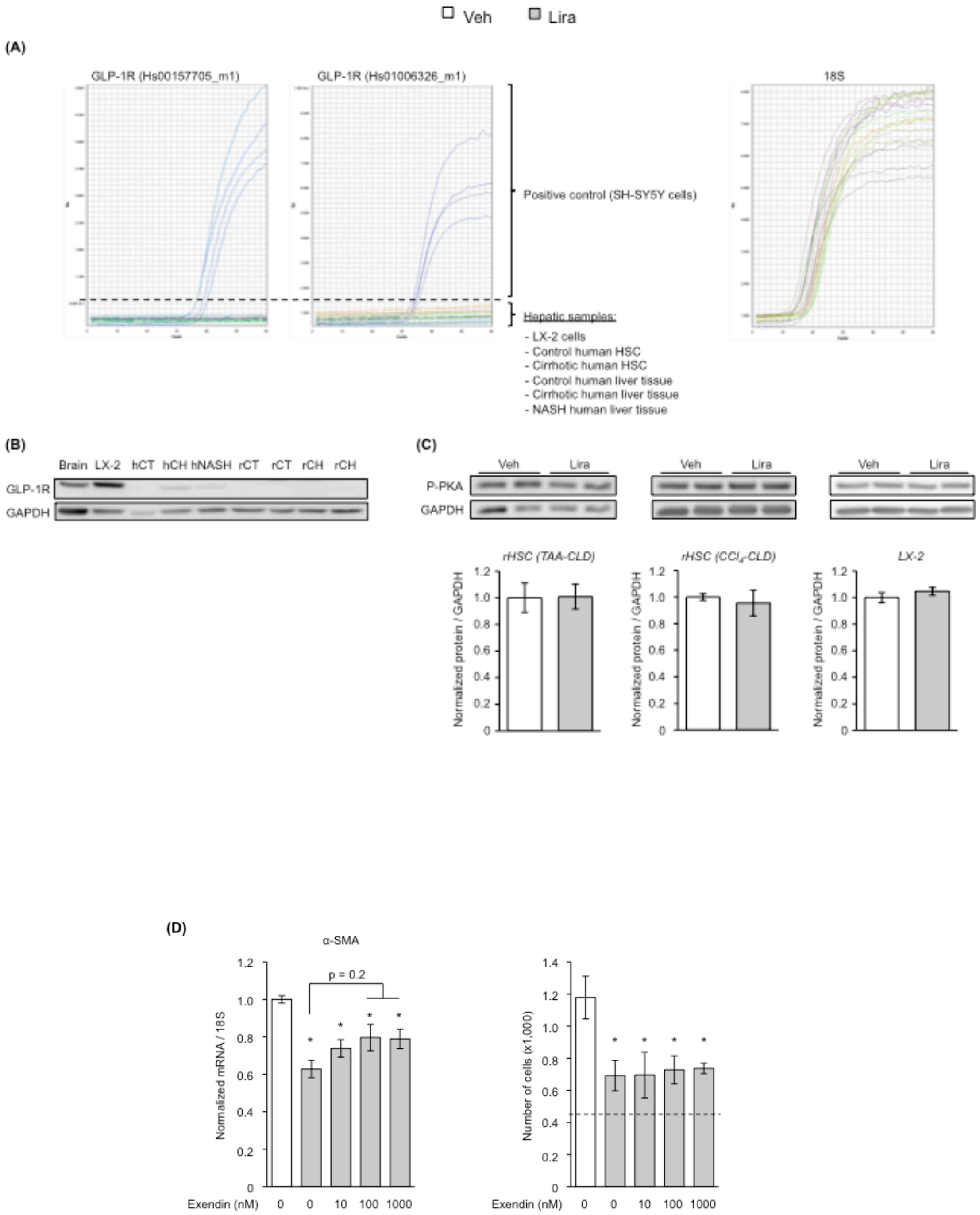

Supplementary figure 5. (a) 60-cycle amplification charts of GLP-1R mRNA expression analyzed in human liver & cells samples using the human cell line SH-SY5Y as positive control. **(b)** Western blot of different hepatic samples using a GLP-1R antibody (Abcam ab189397). From left to right, brain tissue as positive control, LX-2 cells, human liver tissue (control, cirrhotic, NASH) and rat

liver tissue (control, cirrhotic). **(c)** p-PKA protein expression in primary cirrhotic HSC from TAA-CLD (left), CCl_4-CLD (middle) and in LX-2 cells (right) in response to 50μM liraglutide. **(d)** Effects of the GLP-1R antagonist exendin 9-39 on α-SMA mRNA expression (left) and proliferation (right) in LX-2 cells. n=3 per experimental condition. *p<0.05 vs. vehicle.

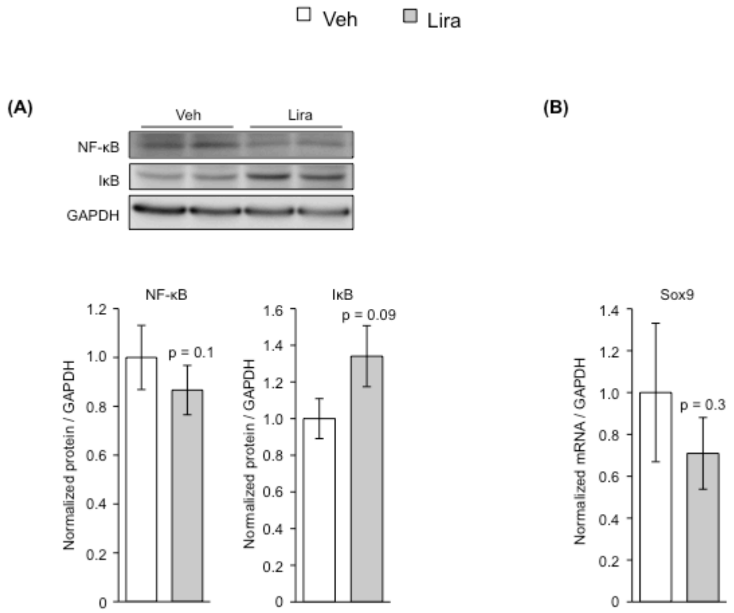

Supplementary figure 6. (a) Protein expression of NF-κB and its inhibitor IκB and **(b)** mRNA expression of Sox9 in livers from TAA-CLD-rats treated with liraglutide, or vehicle. n=8 per group.
